# Nicotinamide Phosphoribosyl Transferase (Nampt) Is a Target of MicroRNA-26b in Colorectal Cancer Cells

**DOI:** 10.1371/journal.pone.0069963

**Published:** 2013-07-29

**Authors:** Chenpeng Zhang, Jinlu Tong, Gang Huang

**Affiliations:** 1 Department of Nuclear Medicine, Ren Ji hospital, School of medicine, Shanghai Jiao Tong University, Shanghai, China; 2 Med-X Research Institute and School of Biomedical Engineering, Shanghai Jiao Tong University, Shanghai, China; 3 Department of Gastroenterology, Shanghai Institute of Gastrointestinal Diseases; Ren Ji Hospital, School of Medicine, Shanghai Jiao Tong University, Shanghai, China; Vanderbilt University Medical Center, United States of America

## Abstract

A number of cancers show increased expression of Nicotinamide phosphoribosyl transferase (Nampt). However, the mechanism through which Nampt is upregulated is unclear. In our study, we found that the Nampt-specific chemical inhibitor FK866 significantly inhibited cell survival and reduced nicotinamide adenine dinucleotide (NAD) levels in LoVo and SW480 cell lines. Bioinformatics analyses suggested that miR-26b targets Nampt mRNA. We identified Nampt as a new target of miR-26b and demonstrated that miR-26b inhibits Nampt expression at the protein and mRNA levels by binding to the Nampt 3′-UTR. Moreover, we found that miR-26b was down regulated in cancer tissues relative to that in adjacent normal tissues in 18 colorectal cancer patients. A statistically significant inverse correlation between miR-26b and Nampt expression was observed in samples from colorectal cancer patients and in 5 colorectal cell lines (HT-29, SW480, SW1116, LoVo, and HCT116). In addition, over expression of miR-26b strongly inhibited LoVo cell survival and invasion, an effect partially abrogated by the addition of NAD. In conclusion, this study demonstrated that the NAD-salvaging biosynthesis pathway involving Nampt might play a role in colorectal cancer cell survival. MiR-26b may serve as a tumor suppressor by targeting Nampt.

## Introduction

The protein nicotinamide phosphoribosyl transferase (Nampt) has several functions. It exists in intercellular (iNAMPT) and extracellular (eNAMPT) forms [1]. In cells, it functions as a rate-limiting enzyme in the salvage pathway for the synthesis of nicotinamide adenine dinucleotide (NAD), which is involved in cell metabolism and proliferation [2]. Extracellular Nampt was first identified as pre-B-cell colony enhancing factor (PBEF), a cytokine-like protein that stimulated early B-cell formation [3]. It was renamed recently as visfatin by Fukuhara et al., who identified it as a visceral fat-derived adipokine that is believed to mimic insulin function [4]. Nampt has drawn significant interest not only in the fields of metabolism and immune response, but also in the field of cancer. A number of cancers show increased expression of Nampt [5], [6], [7], [8], [9], [10], [11], and inhibitors of Nampt offer promising therapeutic applications in cancer [12], [13], [14], [15]. However, the mechanism through which Nampt becomes upregulated is unclear.

MicroRNAs (miRNAs) are a class of small non-coding RNAs. Identified as a new class of gene expression regulators, they target mRNAs for translational repression or degradation [16]. A series of studies have revealed that miRNAs can regulate the expression of a variety of genes, including those important for tumor proliferation, invasion, or metastasis [17], [18], [19]. The usefulness of miRNAs in the treatment of cancer has been demonstrated [20], [21], [22].

In this study, we demonstrated that the NAD-salvaging biosynthesis pathway involving Nampt plays a role in colorectal cancer cell survival. We showed for the first time that Nampt is a target of miR-26b. Moreover, the expression of miR-26b and Nampt was assayed in human samples of colorectal cancer patients and 5 colorectal cancer cells. The roles of miR-26b and Nampt in human cancer development are discussed.

## Methods

### Ethics Statement

This study is approved by the Ethics Committee of Ren Ji Hospital, School of medicine, Shanghai Jiao Tong University. This study was performed in strict accordance with the recommendations in the Guide for the Care and Use of Human Samples from Ren Ji Hospital.

### Cell Lines and Tissue Samples

Colorectal cancer cell lines SW480, SW1116, HT-29, LoVo, and HCT116 were used in this study (obtained from Shanghai Institute of Gastrointestinal Diseases, Ren Ji Hospital, School of medicine, Shanghai Jiao Tong University, China). All cell lines were cultured in RPMI-1640 medium supplemented with 10% fetal bovine serum (FBS) in a 5% CO_2_ humidified incubator at 37°C.

Tissue samples from human colorectal and adjacent normal colorectal tissues were obtained from 18 patients who underwent surgery in Ren Ji Hospital, School of medicine, Shanghai Jiao Tong University (Shanghai, China). None of the patients received chemotherapy or radiotherapy before surgery. Samples were collected and stored at −80°C. The histopathologic diagnoses were evaluated by the hospital’s pathologist using both morphologic criteria and immunocytochemistry. All patients have given their written informed consent (as outlined in PLOS consent form) before tissue collection, and the study was approved by the ethics committees of the Ren Ji Hospital, School of medicine, Shanghai Jiao Tong University.

### CCK-8 Assay

The cell survival rate was examined using Cell Counting Kit-8 (CCK-8) (Dojindo). Cells were plated in 96-well plates at 5000 cells/well in complete medium and cultured for 24 h. The medium was then replaced with RPMI-1640 containing 10% FBS, with or without treatment. After incubation, 10 µL of CCK-8 was added to each well, and the plates were further incubated for 4 h in an incubator. The absorbance at 490 nm was read spectrophotometrically using a microplate reader.

### Apoptosis Assay

Cell apoptosis was determined by the Vybrant apoptosis assay kit #2 (Invitrogen) according to the manufacturer’s protocol. Each sample was evaluated by flow cytometry with a Coulter Epics XL flow cytometer (Beckman-Coulter). Data were processed with Expo 32 cytometer software (Beckman-Coulter). Experiments were done three times in duplicate.

### NAD+ and NADH Quantification

The concentrations of NAD^+^ and NADH in cells were detected using a NAD^+^/NADH Quantification Kit (Bio Vision) according to the manual. The amount of NAD^+^ in each sample was normalized to the protein content for each test sample.

### Prediction of miRNA Targets

Computer-based TargetScan (http://www.targetscan.org/) was used to predict the miRNAs that target Nampt mRNA.

### Knockdown or Overexpression of miR-26b

Expression of miR-26b in cells was knocked down by transfection with miR-26b inhibitor (GenePharma) or increased by transfection with miR-26b mimics (GenePharma). The cells were plated in culture dishes or 24-well plates for 24 h and then transfected with inhibitor or mimics using Lipofectamine 2000 (Invitrogen) for 24 h. After transfection, the cells were subjected to further assays or to RNA/protein extraction.

### RNA Extraction and Real-time RT–PCR

Total RNA was extracted from cultured cells and the colorectal cancer specimens using TRIzol (Invitrogen). The levels of Nampt mRNA and miR-26b were measured by real-time RT-PCR. For detection of Nampt mRNA, reverse transcription was performed using the M-MuLV Reverse Transcriptase System, and real-time PCR was carried out using SYBR Green (Takara) with the ABI Prism 7700 Sequence Detection System (Applied Biosystems). Glyceraldehyde 3-phosphate dehydrogenase (GAPDH) was used for normalization. To measure the miR-26b levels, quantitative real-time RT-PCR analysis was performed using the TaqMan Reverse Transcription Kit and TaqMan MicroRNA Assays Kit (Applied Biosystems) according to the manufacturers’ instructions. A human U6 small nuclear RNA TaqMan probe was used for normalization. Primer sequences (forward and reverse) are listed in Table 1.

**Table 1:** Primer sequences (forward and reverse).

Gene	sequence
Nampt-f	GCCAGCAGGGAATTTTGTTA
Nampt-r	TGATGTGCTGCTTCCAGTTC
GAPDH-f	AGGCCGGTGCTGAGTATGTC
GAPDH-r	TGCCTGCTTCACCACCTTCT

### Western Blotting

An equal number of pelleted cells were washed with ice-cold phosphate-buffered saline (PBS), again pelleted, and resuspended in RIPA buffer. Subsequently, the cells were incubated at 95°C for 5 min and centrifuged in a microcentrifuge for 5 min. Equal amounts of total protein were loaded for electrophoresis in sodium dodecyl sulfate polyacrylamide gels and then transferred to nitrocellulose membranes. The membranes were blocked with 5% TBS-T for 1 h at room temperature and incubated with primary antibody in TBS-T for 1 h at room temperature or overnight at 4°C. After washing 4 times for 5 min each in TBS-T, membranes were incubated with horseradish peroxidase-conjugated secondary antibody (Kangchen Technology) for 1 h at room temperature. After washing 4 times for 5 min, protein bands were visualized through chemiluminescence reaction and exposed to X-ray film. The primary antibody, anti-Nampt antibody (catalog number AP9010c), was from Abgent. Data were processed using the 2-ΔΔCT method.

### Reporter Constructs

The putative miRNA26b-recognition elements from the Nampt gene and corresponding mutants were cloned into the 3′-untranslated region (UTR) of the luciferase gene in the pEZX-MT01 luciferase vector (GeneCopoeia Inc.). This expression clone contained the Nampt (Accession: NM_005746.2) 3′-UTR sequence inserted into the pEZX-MT01 vector downstream of a firefly luciferase gene under the control of an SV40 promoter. The pEZX-MT01 vector also contained the Renilla luciferase gene under the control of a CMV promoter. Firefly luciferase activity was normalized to Renilla luciferase activity. The mutant clones for Nampt were constructed. The seed binding region (5′-UACUUGAA-3′) was changed to 5′-UACUUACA-3′ (2-bp replacement). All constructs were confirmed by DNA sequence analysis.

### Luciferase Reporter Assay

SW480 cells were cultured in 24-well plates and transfected with negative control mimic (NC) or miR-26b mimics (30 nM or 60 nM; GenePharma) using Lipofectamine 2000 (Invitrogen). Following a second transfection with a miR-26b reporter construct or its mutant (50 ng), SW480 cells was incubated for 24 h. Luciferase assays were performed using the Dual-Light Combined Reporter Gene Assay System (Promega) and Promega Turner TD-20/20 Luminometer.

### Transwell Assay

Cell migration was performed by transwell assay (BD Biosciences) according to the manufacturer’s instructions. Control miRNA or mimics of miR-26b transfected cells were harvested 24 h after transfection. Then 3.0×10^5^ transfected cells or untreated cells in serum-free medium were added to each upper insert pre-coated with Matrigel matrix. To the matched lower chamber, 500 µL of 10% FBS medium was added. After incubation, non-invasive cells were removed from the upper surface of the transwell membrane with a cotton swab, and the invasive cells on the lower membrane surface were fixed in methanol, stained with 0.1% crystal violet, photographed, and counted.

### Statistical Analysis

The experiments were repeated 3 times. Data are expressed as mean ± SD. For comparison of 2 groups, a two-tailed, unpaired t-test was used. A value of p<0.05 was considered statistically significant. Statistical tests were performed using State Software 12.0 (Stata Corp).

## Results

### Nampt-specific Chemical Inhibitor FK866 Inhibits NAD Synthesis and Cell Survival

Increased Nampt expression has been reported in primary colorectal cancer [5], [6], [7]. FK866 is a Nampt-specific chemical inhibitor that inhibits the NAD-salvaging biosynthesis pathway by binding at the nicotinamide-binding site of Nampt, thereby depleting cells of NAD [23], [24]. Reportedly, FK866 is well tolerated, and it significantly inhibits cell growth in some type tumors [13], [15]. However, the effect of FK866 in colorectal cancer has not been documented.

**Figure 1:**
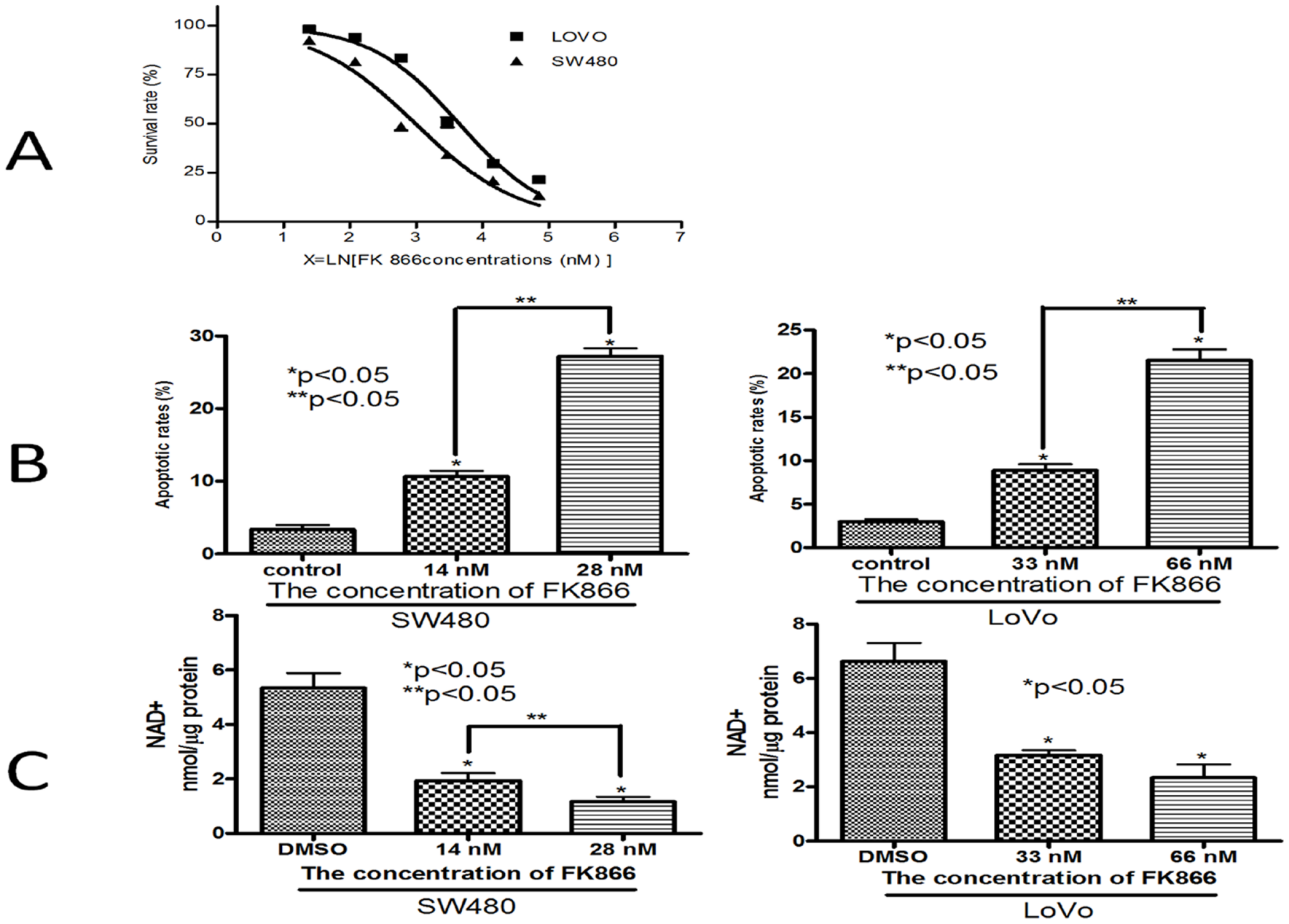
Nampt inhibitor FK866 reduced cell survival rate and the NAD level in a dose-dependent manner in SW480 and LoVo cell lines. A. Cells were treated with different concentrations of FK866 for 72 h and then evaluated by using Cell Counting Kit-8. The IC_50_ values were calculated. Each assay was repeated at least 3 times. B. The quantification of apoptotic cells induced by FK866 was confirmed by flow cytometry analysis. The sub-G1 contents were designated apoptotic cells. *: versus negative control group; **: compared with 2 treatment groups. C. NAD levels in colorectal cancer cells were measured after exposure to FK866. Each vertical bar represents the mean ± SD of triplicate determinations. For comparison of 2 groups, a two-tailed, unpaired t-test was used.

We analyzed the effect of FK866 on the cell survival rate of human colorectal cancer SW480 and LoVo cells by CCK-8 assay and flow cytometry. The results showed that FK866 significantly promoted apoptosis of SW480 and LoVo cells in a dose-dependent manner. The IC_50_ of FK866 was 14.3 nM for SW480 cells and 32.7 nM for LoVo cells, according to the CCK-8 assay (Figure 1A). Flow cytometry studies showed that exposing SW480 and LoVo cells to different concentration of FK866 for 3 days increased the percentage of early-stage apoptotic cells from 3.37±0.83% to 27.27±1.49% (SW480) and 3.00±0.37% to 21.53±1.76% (LoVo) (Figure 1B).

We next determined the effect of FK866 on NAD biosynthesis in cells using the NAD^+^/NADH Quantification Kit. FK866 exposure for 3 days decreased the level of NAD from 5.33±0.96 to 1.17±0.31 nM/µg (SW480) and 6.63±1.16 to 2.33±0.85 nM/µg (LoVo) (Figure 1C). These results suggested that Nampt-mediated NAD-salvaging biosynthesis plays a critical role in colorectal cancer cell survival and that FK866 has anti-carcinogenic properties in colorectal cancer cells.

### MiR-26b Inhibits the Expression of Nampt via Binding to its 3′-UTR

By computer-based sequence analysis using TargetScans detection software (http://www.targetscan.org/), Nampt was predicted to be a potential target of miR-26b. The seed sequence of mature miR-26b was conserved among human, chimpanzee, rhesus, and bush baby species, among others. Therefore, miR-26b was selected for further biological characterization.

**Figure 2:**
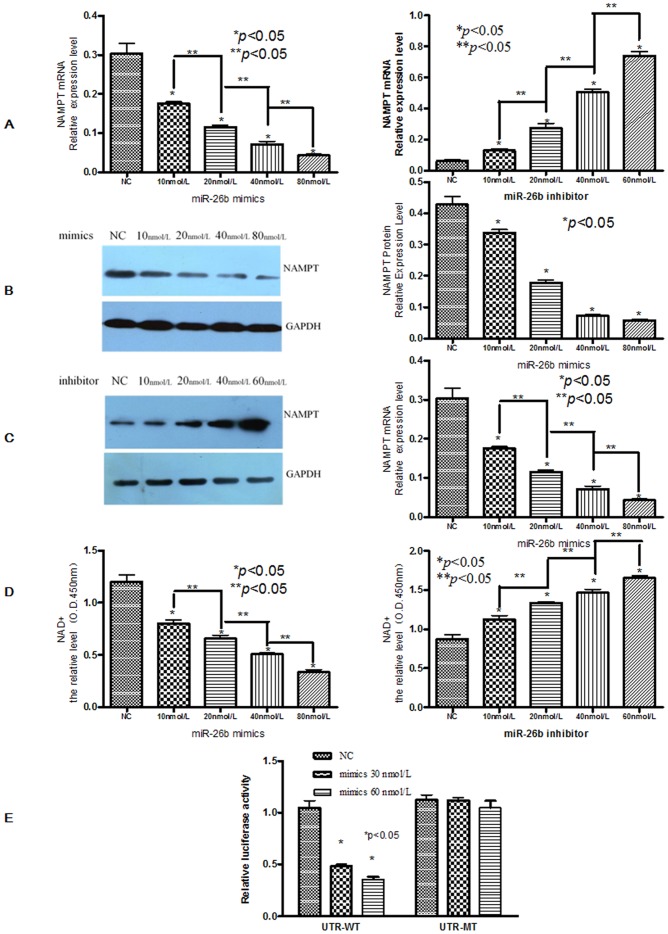
The effects of miR-26b on Nampt expression. A. Quantitative real-time PCR of Nampt in SW480 cell lines after transfection with different concentrations of miR-26b mimics or inhibitors. B and C. Western blotting of Nampt in SW480 cell lines after transfection with different concentrations of miR-26b mimics or inhibitors. D. The relative levels of NAD in SW480 cell lines after transfection with different concentrations of miR-26b mimics or inhibitors. E. Luciferase reporter assays validating the direct interaction of miR-26b with the 3′-UTR of Nampt. Nampt-3′-UTR-pEZX (or Nampt-3′-UTR-mut-pEZX) was cotransfected with miR-26b mimic or negative control into SW480 cells. *: versus negative control group; **: compared with 2 treatment groups. Each vertical bar represents the mean ± SD of triplicate determinations. For comparison of 2 groups, a two-tailed, unpaired t-test was used.

By miR-26b mimics, overexpression of miR-26b suppressed Nampt mRNA (Figure 2A left) and protein (Figure 2B) levels in SW480 cells, as detected by RT-PCR and western blot analysis, respectively. Meanwhile, miR-26b mimics also suppressed the expression level of NAD+, which was measured by the NAD^+^/NADH Quantification Kit (Figure 2D left). On the other hand, transfection with miR-26b inhibitor increased Nampt mRNA (Figure 2A right) and protein (Figure 2C) levels, and miR-26b inhibitor also increased the expression level of NAD+ (Figure 2D right).

To confirm that Nampt is the target of miR-26b, the effect of transfection with miR-26b mimics on the reporter activity of wide type and mutated pEZX-Nampt was examined. pEZX-Nampt is a dual-luciferase reporter construct containing a wide type or mutated segment of the Nampt 3′-UTR with the miR-26b recognition sequence immediately downstream of the firefly luciferase reporter (Figure S1A). A diagram of Nampt with wild type or mutated sequences in the potential miR-26b binding site is shown in Figure S1B. The sequence maps of wild type and mutated Nampt obtained by gene sequencing are shown in Figure S1C. As shown in Figure 2E, transfection with different concentration of miR-26b mimics decreased the firefly luciferase reporter activity of wild type pEZX-Nampt by approximately 51.7%±3.2% and 64.3%±4.0%, respectively. The induction of reporter activity by overexpression of miR-26b by miR-26b mimics, however, was not detected when the miR-26b recognition sequence in the 3′-UTR of Nampt mRNA was mutated (Figure 2E). Therefore, the results from Figures 2 confirm that Nampt is a target of miR-26b.

### The Expression Levels of miR-26b and Nampt in Colorectal Cancer Tissues and Cell Lines

**Figure 3:**
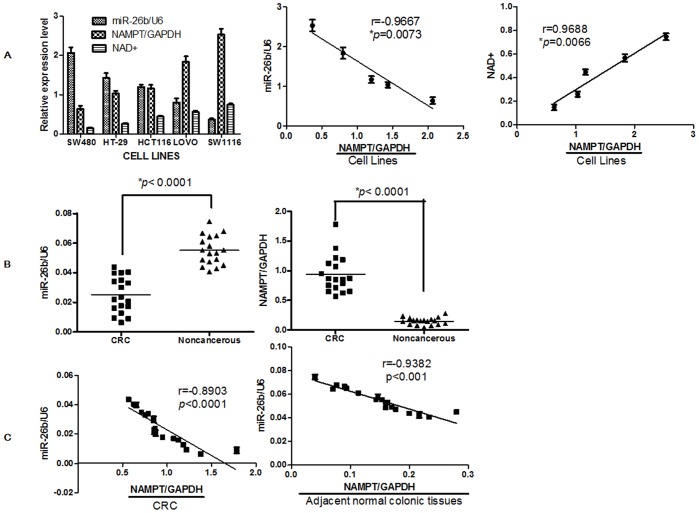
Nampt mRNA levels were inversely correlated with miR-26b levels in colorectal cancer cell lines and patient samples. A. miR-26b, Nampt mRNA and NAD+ levels were assayed in colorectal cancer cell lines. The expression of miR-26b was inversely correlated with Nampt expression in colorectal cancer cell lines; the expression of NAD+ was positive correlated with Nampt expression in colorectal cancer cell lines (A right). B. miR-26b and Nampt mRNA levels were assayed in 18 surgical specimens of human colorectal cancer tissues and adjacent normal colorectal tissues. Significantly upregulated Nampt levels in colorectal cancer tissues are shown relative to Nampt levels in adjacent normal colorectal tissues (fold changes >6); significantly downregulated miR-26b levels in colorectal cancer tissues are shown relative to miR-26b levels in adjacent normal colorectal tissues (fold changes >2). C. The expression of miR-26b was inversely correlated with Nampt expression in colorectal cancer tissues (C left), and adjacent normal colorectal tissues (C right). Nampt mRNA levels were assayed by real-time RT-PCR and normalized to GAPDH. The miR-26b levels were assayed by real-time RT-PCR and normalized to U6. Each vertical bar represents the mean ± SD of triplicate determinations. For comparison of 2 groups, a two-tailed, unpaired t-test was used.

The basal expression levels of miR-26b and Nampt mRNA were measured by qRT-PCR in colorectal cancer cell lines (HT-29, SW480, SW1116, LoVo, and HCT116), and the basal expression levels of NAD+ also were measured by NAD^+^/NADH Quantification Kit in cell lines. SW480 cells had the lowest Nampt expression and NAD+ level, and the highest miR-26b level, followed by HT-29, HCT116, LoVo, and SW116 cells (Figure 3A left). A significant inverse correlation between the expression of miR-26b and Nampt mRNA was observed, and a significant positive correlation between NAD+ and Nampt mRNA was also observed (Figure 3A right).

In addition, we extended our investigation to samples from colorectal cancer patients. Our results showed that Nampt expression was significantly increased in cancer tissues (6.3-fold) when compared with that in the paired adjacent normal tissues in the panel of 18 colorectal cancer patients (Figure 3B right), which was consistent with others’ findings [5], [6], [7]. In addition, we found the cancerous tissue showed a notable loss of miR-26b (∼45.45%) as compared with the adjacent normal colorectal cancer tissues in the panel of 18 colorectal cancer patients (Figure 3B left), which has not been reported before. We observed an inverse correlation between miR-26b and Nampt expression in cancerous tissues (Figure 3C left) and adjacent normal tissues (Figure 3C right).

### MiR-26b Inhibits Cell Survival and Invasion, and this can be Partially Abrogated by the Addition of NAD

The Nampt is known to regulate a variety of biological activities, including cell survival and invasion. Since miR-26b is believed to target Nampt, the effect of miR-26b on cell survival and invasion was investigated. Furthermore, we co-transfected cells with miR-26b mimic, then 1.0 mM/L NAD+ (maximally effective) was added to determine whether the effects of miR-26b are mediated by Nampt-mediated NAD biosynthesis.

**Figure 4:**
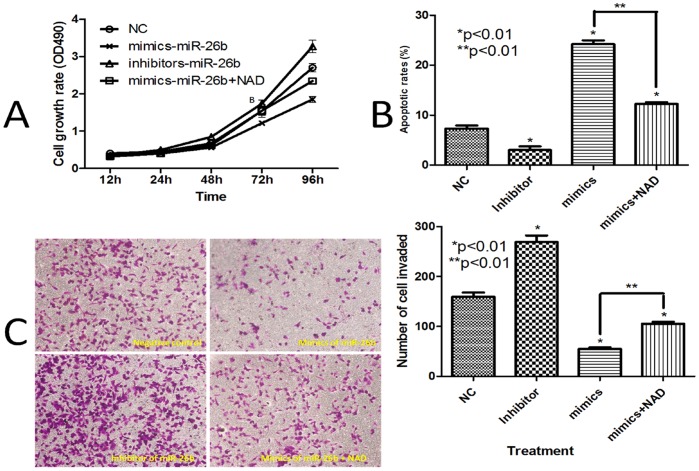
Effects of miR-26b and NAD+ on the cell survival and invasiveness of colorectal cancer cells. A. LoVo cells were treated with miR-26b mimics et al., and cell viability was determined using Cell Counting Kit-8. B. The quantification of apoptotic cells induced by miR-26b mimics (with and without NAD) or miR-26b inhibitor was confirmed by flow cytometry analysis. The sub-G1 contents were designated apoptotic cells. C. Representative micrographs of cell invasion assays (left) and the quantification (right) from different treatments (*p<0.01). The stained invasive cells were photographed under an inverted light microscope (100× magnification). *: versus negative control group; **: compared with 2 treatment groups. Each vertical bar represents the mean ± SD of triplicate determinations. For comparison of 2 groups, a two-tailed, unpaired t-test was used.

Using the CKK-8 assay, cells treated with miR-26b inhibitor had the highest cell proliferation, followed by cells treated with negative control, miR-26b mimics plus NAD, and miR-26b mimics alone (Figure 4A). A 21.1% increase in cell growth was observed in LoVo cells 4 days after transfection with miR-26b inhibitor, when compared to growth in the negative control. However, transfection with miR-26b mimics suppressed the growth of LoVo cells by 31.48% compared to the negative control. Transfection with miR-26b mimics plus NAD increased the growth of LoVo cells by 26.85% compared to the group transfected with only miR-26b mimics.

The flow cytometry assay results are shown in Figure 4B. Exposing LoVo cells to miR-26b inhibitors for 4 days significantly decreased the percentage of early-stage apoptotic cells (3.03±0.72% compared to the 7.33±0.60% in the negative control group). Transfection with miR-26b mimics and NAD remarkably decreased the early-stage apoptotic rate (12.23±0.35% compared to 24.2±0.79% in the group transfected only with miR-26b mimics).

Of interest, miR-26b suppression affected not only cell survival but also invasion (Figure 4C). Transfection with miR-26b inhibitor remarkably increased the invasion efficiency of LoVo cells (1.7-fold) according to the Transwell assay. Transfection with miR-26b mimics, however, decreased the invasion efficiency of LoVo cells to 34.53%, and this was partially rescued by the addition of NAD (increased to 66.10%). Results from Figure 4 therefore indicate that miR-26b inhibited cell survival and invasion in LoVo cells. The addition of NAD partially abrogated this effect.

## Discussion

Studies have shown that Nampt is significantly increased in primary colorectal cancer [5], [6], [7] and other cancers [8], [9], [10], [11]. Thus, Nampt may be a good biomarker of malignant potential and stage progression [1], [8], [25], [26]. Nampt-mediated NAD biosynthesis plays a critical role in cell metabolism, survival functions, and stress resistance through sirtuins and other NAD-consuming regulators [1]. The Nampt-specific chemical inhibitor FK866 depletes cells of intracellular NAD by binding at the nicotinamide-binding site of Nampt and inhibiting the NAD salvage pathway [23], [24]. In cancer cells, FK866 displays anti-tumor [27], anti-metastatic [15], and anti-angiogenic activities [28] and a chemo-sensitizing effect [27]. In contrast, some studies showed non-tumorigenic cells were unaffected by FK866 [29], [30]. These might be explained by the fact that cancer cells require higher NAD+ levels to accommodate with high metabolic rate than normal cells. Consequently, there is a difference in dose-response between malignant and normal cells as for the levels of the cellular NAD+ content. Therefore, FK866 is well tolerated, and it significantly inhibits tumor growth in vitro and in vivo. These results are supported by many researches [12], [13], [14], [15]. In this study, we first showed that FK866 significantly inhibited cell survival and reduced NAD levels in SW480 and LoVo cells, which suggests that the Nampt-mediated NAD biosynthetic mechanism is active in colorectal cancer cells. Interesting, there was little correlation between the decrease in NAD+ and the increase in apoptosis using FK866 according to our results (r = −0.8544, *p* = 0.3479). There might be two different conjectures as for the result. Firstly, there might be a threshold effect because of the high concentration of FK866. Secondly, Thakur, B. K. reported FK866 is able to induce apoptosis in leukemic cells by indirectly activating the tumor-suppressor protein p53 [13], and this pathway might also work in colorectal cancer cells. Though there was little correlation between NAD+ and apoptosis according to our results, this would not affect the fact that FK866 might be a promising agent for colorectal cancers. And the molecular mechanisms responsible for the high expression of Nampt in colorectal cancer are not fully understood.

Recently, it was reported that inappropriate expression of miRNAs contributes to the pathogenesis of cancer. Decreased levels of miR-26b are observed in a wide range of tumors [17], [31], [32], [33], [34], [35], [36], [37]. In our studies, we showed that the expression of miR-26b was significantly downregulated in 18 cancerous samples of colorectal cancer patients. In colorectal cancer patients, miR-26b was 2.2-fold higher in adjacent normal tissues than in cancerous tissues (p<0.0001). We originally identified Nampt as a target of miR-26b by bioinformatics analyses, luciferase reporter assays, and faction assay by transfection of miR-26 mimics or inhibitors. Overexpression of miR-26b by transfection of miR-26 mimics significantly decreased Nampt mRNA and protein levels in vitro. Our study found that miR-26b mimics are more effective in apoptosis than in the proliferation assays. The results consistent with other studies that inhibition of Nampt caused general cytotoxicity [13]. Knockdown of miR-26b by transfection of miR-26b inhibitors significantly increased Nampt mRNA and protein levels. This indicates that miR-26b regulates Nampt primarily through translational repression. Meanwhile, in our results the regulatory pathways of miR-26b were associated with invasiveness and metastasis in colorectal cancer cells. Ma et al. showed that overexpression of miR-26b inhibited in vivo tumor growth in mice injected with LoVo cells [38]. Furthermore, we found that the inhibition of cell survival and invasion by overexpression of miR-26b could be partially rescued by the addition of NAD. This result indicates that miR-26b inhibition of tumor cell survival and invasion involves Nampt-mediated NAD biosynthesis. Hence, our findings provide insight into the function of miR-26b in regulating colorectal tumorigenesis. In addition, given that Nampt functions in metabolism and immune responses, miR-26b may also affect these processes by attenuating the translation of Nampt. This suggests a mechanistic explanation for the downregulation of miR-26b in diabetic mice [39].

However, the mechanism by which miR-26b and Nampt regulate tumorigenesis may not be so simple. When the concentration of NAD+ was maximally effective, the rescue was not fully recovered. The alternative pathway might be possible involvement. First, recent studies indicate that miR-26b has many other direct targets, such as EphA2 [40], SLC7A11 [32], Lef-1 [41], pRb protein [42], and COX-2 [36]. Second, miRNAs other than miR-26b might target Nampt expression. For example, miR-182 downregulated Nampt expression in Tat-induced HIV-1 long terminal repeat (LTR) transactivation [43]. Third, although it reported that the regulation of the glucose stimulated insulin secretion and mTORC1 and ERK1/2 signal pathway are involved in Nampt-media NAD salvage synthesis [12], [44], other signaling pathways involving Nampt, such as PI3K/Akt [45] and STAT3 [46], [47], also participate in tumor formation, transformation, and angiogenesis. Taken together, the miR-26b-Nampt-NAD signaling pathway contributes to colorectal cancer cell survival and invasion, but it is not the only pathway.

### Conclusion

In summary, our present study suggests that miR-26b negatively regulates Nampt expression at the posttranscriptional level by binding to its 3′-UTR. Additionally, miR-26b inhibited tumor growth and invasion, and this effect was partially abrogated by the addition of NAD. We also found that levels of miR-26b in patient colorectal tumor tissues were much lower than in adjacent normal tissues, and that miR-26b expression inversely correlated with the expression of Nampt mRNA in 5 colorectal cancer cell lines and patient samples. These results indicate that our results are clinically relevant: miR-26b overexpression and inhibition of the Nampt-NAD signaling pathway may be a rationale for therapeutic interventions in colorectal cancer in the future.

## Supporting Information

Figure S1 Luciferase reporter assays validating the interaction between miR-26b and Nampt. A. Construction of the pEZX-MT01 luciferase reporter (Luc-Nampt 3′-UTR). hLuc, firefly luciferase reporter gene; hRLuc, Renilla luciferase reporter gene. Firefly luciferase expression is regulated by binding of the miRNA to the 3′-UTR target sequence. Firefly luciferase activity was normalized to Renilla luciferase activity. B. Diagram of Nampt with wild type and mutated sequences in the potential miR-26b binding site. C. Sequence maps of wild type and mutated Nampt sequences established by gene sequencing.
(TIF)
