# Supplementary figures and images for: Nicotinamide Phosphoribosyl Transferase (Nampt) Is a Target of MicroRNA-26b in Colorectal Cancer Cells
Source: PLoS One. 2013 Jul 29;8(7):e69963. doi: 10.1371/journal.pone.0069963 (PMC3726743; doi:10.1371/journal.pone.0069963)

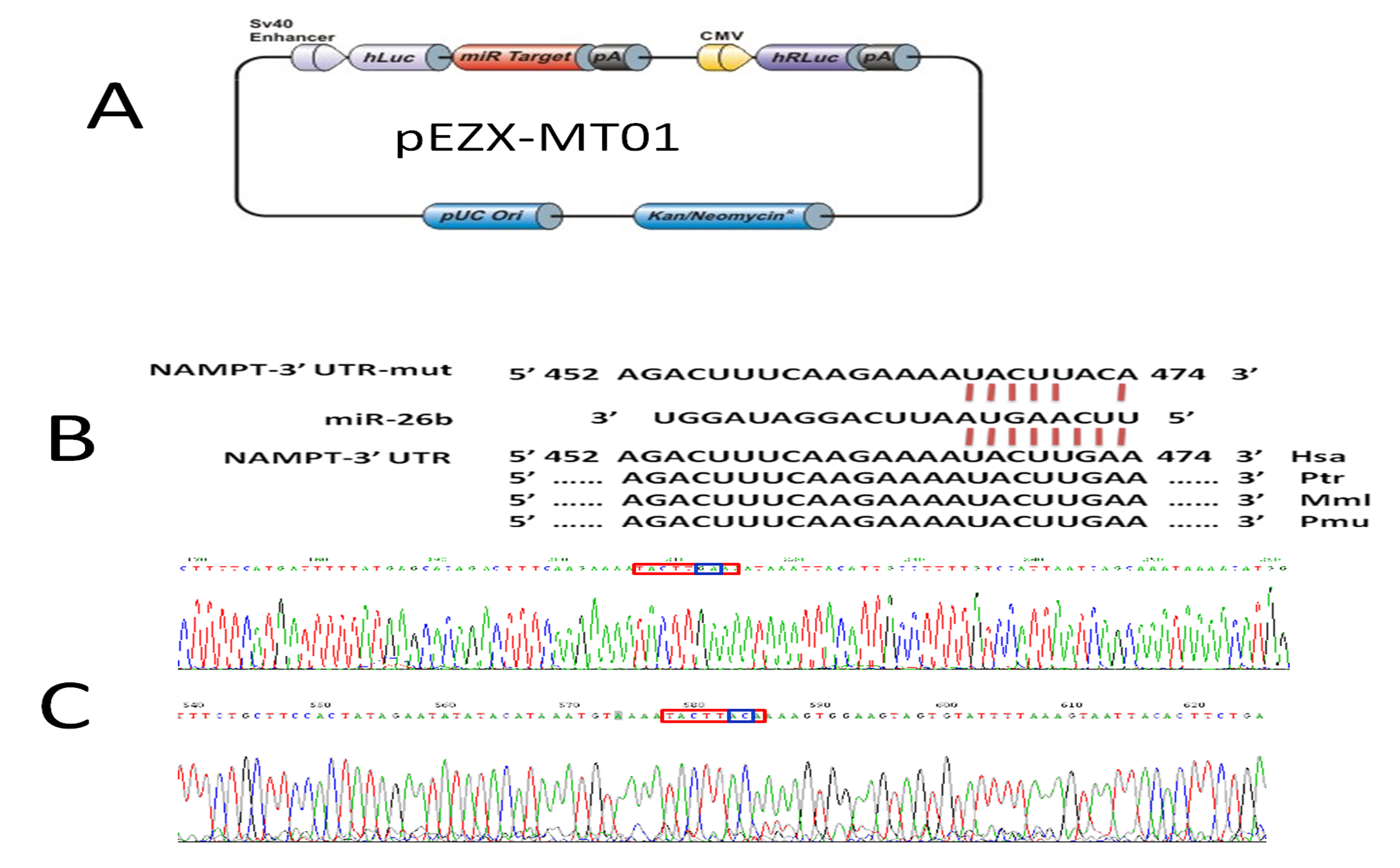

Supplement: Figure S1 — Luciferase reporter assays validating the interaction between miR-26b and Nampt. A. Construction of the pEZX-MT01 luciferase reporter (Luc-Nampt 3′-UTR). hLuc, firefly luciferase reporter gene; hRLuc, Renilla luciferase reporter gene. Firefly luciferase expression is regulated by binding of the miRNA to the 3′-UTR target sequence. Firefly luciferase activity was normalized to Renilla luciferase activity. B. Diagram of Nampt with wild type and mutated sequences in the potential miR-26b binding site. C. Sequence maps of wild type and mutated Nampt sequences established by gene sequencing. (TIF) [file pone.0069963.s001.tif]
